# Evaluation of the impact of COVID-19 pandemic on hospital-acquired infections in a tertiary hospital in Malaysia

**DOI:** 10.1186/s12879-023-08770-3

**Published:** 2023-11-09

**Authors:** Rehab Ismaeil, Abdul Rahman Fata Nahas, Norhidayah Binti Kamarudin, Usman Abubakar, Mohamad Basri Mat-Nor, Mohamad Haniki Nik Mohamed

**Affiliations:** 1 Department of Pharmacy Practice, Kulliyah of Pharmacy, International Islamic University Malaysia (IIUM), Jalan Sultan Ahmad Shah, 25200 Kuantan, Pahang, Malaysia; 2 Department of Medical Microbiology, Kulliyyah of Medicine, International Islamic University Malaysia, 25200 Kuantan, Malaysia; 3 Department of Clinical Pharmacy and Practice, College of Pharmacy, QU Health, Qatar University, 2713 Doha, Qatar; 4 Department of Intensive Care, Kulliyyah of Medicine, International Islamic University Malaysia, 25200 Kuantan, Malaysia

**Keywords:** COVID-19, Hospital acquired infection, Prevalence, Infection prevention and control

## Abstract

**Background:**

Infection prevention measures are the gold standard for preventing the spread of hospital-acquired infections (HAIs). COVID-19 pandemic caused major disruptions in infection prevention measures, and this has implications on the rate of HAIs. This study assessed the impact of COVID-19 pandemic on the rate and the types of HAIs at Sultan Ahmed Shah Hospital.

**Method:**

This is a retrospective cohort study that compared the rate of HAIs from April to October 2019 (pre COVID period) and April to October 2020 (during COVID period). Data was collected through the review of patients’ electronic medical records.

**Results:**

There were a total of 578 patients included in the selected wards during the pre- and during the pandemic. Thirty-nine episodes (12.1%) of HAIs were report in the pre COVID period and 29 (11.3%) during COVID-19. In both periods, hospital-acquired pneumonia (HAP) was the most frequent HAI among the patients. There was a rise in catheter-associated bloodstream infections (CLABSI) (0.8%) and ventilator associated pneumonia (VAP) (1.1%) during the COVID-19 period. The most common bacteria were *methicillin-resistant Staphylococcus aureus* (MRSA) (28.2%) and *Enterococcus faecalis* (17.9%) in the Pre COVID-19 period, and *Pseudomonas aeruginosa* (27.6%) and *Stenotrophomonas maltophilia* (6.9%) during COVID-19.

**Conclusion:**

Our research concluded that the rates of HAIs during the COVID-19 pandemic were not significantly impacted by the improved in-hospital infection prevention efforts to control the pandemic. There is need for further efforts to promote adherence to preventive practices.

## Introduction

Hospital acquired infections (HAIs) are one of the most prevalent issues affecting hospitalized patients and dramatically increase mortality and morbidity [1]. HAIs impose a substantial financial burden due to the fact that they prolong patient hospitalizations and increase drug consumption [2]. According to the World Health Organisation (WHO), a huge number of people globally are affected by HAIs. Patients with at least one type of HAI account for 7% of patients in high-income countries and 10% of patients in developing countries [3]. Implementing effective HAI surveillance programmes and appropriate infection prevention procedures can reduce infection rates [4, 5]. However, pandemics bring new responsibilities that demand planning and regular interactions [6]. The significance of infection prevention and control (IPC) in preventing the spread of disease has been underscored by COVID-19 pandemics.

The global healthcare system has faced significant challenges as a result of COVID-19 pandemic [7]. Healthcare facilities were struggling to manage COVID-19 outbreak while also providing existing healthcare services [7–9]. Hospitals have implemented extensive IPC strategies in response to rising caseloads [8, 9]. However, the impact of COVID-19 preventive measures on HAIs is still inadequate [10]. Multiple studies claim that the COVID-19 pandemic influenced the emergence of HAI throughout time [4, 11]. Nevertheless, others indicate that the wide spread of COVID-19 led to a shift in public behavior and a greater understanding of the need for IPC [12, 13]. The use of contact precautions and implementing preventive measures based on the COVID-19 pandemic had a beneficial indirect impact on preventing HAIs [12–14].

In Malaysia, HAIs are a major problem in the healthcare system. Pneumonia, urinary tract infections (UTIs), and surgical site infections (SSIs) accounted for the majority of HAIs, with a prevalence rate of 13% [15, 16]. The strain on the healthcare system during COVID may have indirectly contributed to disruption of conventional healthcare services and compromising patient care [17, 18]. Intensive IPC measures have been implemented as a result of COVID-19 pandemic to stop the spread of infections [17–19]. However, the impact of COVID preventive measures on the rate of HAIs in Malaysia is still unknown. We hypothesized that the deployment of extensive protective preventing measures during the COVID-19 pandemic affected the prevalence of HAIs. So, the aim of this study is to evaluate the impact of COVID-19 pandemic on the rate and the type of HAIs and investigate patterns of microorganisms involved in HAI pre- and during the pandemic at the Sultan Ahmed Shah Medical Centre (SASMEC) Hospital in Malaysia.

## Methodology

### Study design

This was a retrospective cohort study conducted in a tertiary care hospital located on the eastern coast of Malaysia. The study involved two periods, including the period pre-COVID-19 (April to October 2019) and the period during COVID-19 (April to October 2020).

### Study setting and population

The study was conducted in the medical, surgical, and critical/intensive care unit (CICU) wards in SASMEC. SASMEC is a tertiary hospital located on the Eastern Coast of Malaysian Peninsular. It provides a range of services including medical, surgery, pediatrics, orthopedic, neuralgy, cardiology and urology. Adult Patients hospitalised in the selected wards during both periods were included. Those hospitalized for at least 48 h were included in the study. Patients with incomplete medical records, those who were transferred to another hospital or dead, and COVID-19 patients were excluded.

### Data collection

Data was collected through the review of patients’ electronic medical records. The following information was gathered and extracted from patient medical records including: patient demographics (age, gender), clinical data features (gender, age, diagnosis, and comorbidities), information on hospitalization (date of admission, date of discharge), presence of HAI during hospital admission and type of HAI, and microorganisms isolated. In order to ascertain the HAIs, all hospitalised patients were assessed. Data on HAIs was gathered by a specialised infection control team that included a certified nurse and an expert infection control physician. They were responsible for gathering data on HAIs. After a patient was admitted to the hospital, paraclinical testing was performed. The team maintained updated on any changes in the patient's clinical condition continuously. The head nurses provided daily reports, these reports indicate observations of significant signs like fever, changes at the surgical site that signal infection, and adjustments to antibiotic regimens, as well as feedback from the ward physicians regarding their assessment of potential HAIs. Furthermore, the daily culture results from the lab were received to identify any possible infection. Then the standardized data collection form was filled out for each infected patient [20]. This form was approved and submitted to the infection control unit in the hospital. The diagnostic criteria for diagnosing HAIs were developed in accordance with Malaysia's Ministry of Health (MOH) guidelines. These criteria were based on the CDC definition of Nosocomial Infections [21, 22]. All HAI types that occurred throughout the study period were recorded and categorised, including device-associated HAIs, SSIs, UTIs, and hospital- acquired pneumonia (HAP). HAIs are infections that first manifest 48 h or more after being hospitalised or within 30 days of having surgery [1, 20]. The study protocol was approved by the IIUM Research Ethics Committee with a reference number of IREC 2022–078, and the hospital SASMEC approved the study (IIUM/413/013/14/11/1/IISR 22–11-01).

### Statistical analysis

The data was analyzed using the IBM Statistical Package for the Social Sciences (SPSS) version 26 0.0. Descriptive statistics were performed and reported using mean and standard deviation for continuous variables and frequency and percentage for categorical variables. Pearson Chi square test was used to determine the difference in the rate and types of HAIs between the two periods. The overall prevalence of HAI was calculated as the percentage of infected patients out of the total number of hospitalised patients in each period. Percentage change (((new value − old value)/old value) ∗ 100) was used to describe changes in the HAI rates during and pre-COVID-19 [23]. Statistical significance was defined as a *p* value ≤ 0.05. Multivariable logistic regression was performed to evaluate the potential association between the possible risk factor and HAI acquisition. confidence interval (CI) of 95% and significance of *P* < 0.05 were used.

## Results

A total of 578 patients fulfilled the inclusion criteria, including 321 patients in pre-COVID-19 and 257 during COVID-19. The majority of patients were males (59.5%) in the pre COVID-19 and (55.1%) during COVID-19. The mean age of the patients was 59.5 years and 62.0 years pre and during COVID-19 respectively. Hypertension was the most frequent underlying disease in both periods it was (59.5%) in the pre COVID-19 and (62.8%) in the during COVID-19, followed by diabetes, cancer, and coronary heart disease. The proportion of patients having a medical device decreased during the pandemic period, particularly with the use of urinary catheters (4.3%) and mechanical ventilation (3.1%). As well as there was a decline in the number of surgeries during COVID-19 (2.2%) compared to pre-COVID-19 (8.4%) (Table 1).

**Table 1:** Characteristics of the patients admitted to the ICU, Medical and surgical wards in SASMEC hospital pre and during COVID-19 periods

Parameters	Pre COVID Frequency (%) *n* = 321	During COVID Frequency (%) n = 257
**Age** year (mean, SD)	59.56 (12.9**)**	62.08 (13.6)
**Gender**		
Male	190 (59.2%)	177 (55.1)
Female	131(40.8%)	80 (31.2)
**Comorbidities**		
Hypertension	191 (59.5%)	160 (62.8)
Diabetes mellitus	135 (42.1)	116 (45.1)
Coronary heart disease	34 (10.6%)	25 (9.8)
Chronic kidney disease	26 (8.1%)	17 (6.6)
Chronic liver disease	14 (4.4%)	16 (6.2)
Cancer	67 (20.9%)	41 (12.8)
**LOS** mean (SD)	7.9 (5.82)	7.7 (5.8)
**Invasive procedures**		
Urinary catheter	21 (6.5%)	11 (4.3)
Intubation	15 (4.7%)	13 (5.3)
Mechanical ventilation	13 (4%)	8 (3.1)
CVC	6 (1.8%)	4 (1.5)
**Surgery during admission**	27 (8.4)	7 (2.2)
**Ward of admission**		
Surgical	146(45.5%)	117 (45.5)
Medical	96 (29.9%)	98 (38.1)
ICU	79 (24.6%)	42 (16.3)

*ICU* intensive care unit, *CVC* central venous catheter, *LOS* length of stay

Overall, 46 HAI episodes were identified in 39 patients in the pre-COVID-19 period, corresponding to a prevalence of 12.1%. The rate of HAIs during the COVID-19 period was 11.3%, with 32 HAIs episodes reported in 29 patients. HAP was the most common infection in the period pre-COVID-19 (8.1%) and during COVID-19 period (7.4%). There was no statistically significant difference (*p *= 0.75) in the rate of HAP between the two periods. Similarly, there was no significant difference in the rate of UTIs (1.6% versus 1.1%, *p* = 0.69) or SSIs (1.2% versus 0.8%, *p* = 0.57) between the two periods. The rate of ventilator associated pneumonia (VAP) was 0.9% in the pre COVID-19 and 1.2% during COVID-19 (*p* = 0.78). The rate of catheter-associated blood stream infection (CLABSI) was (0.62% versus 0.81%, *p* = 0.82) in the pre and during COVID 19 respectively (Table 2).

**Table 2:** Type and frequency of all of HAIs in patient admitted to the ICU, Medical and surgical wards in SASMEC hospital pre and during COVID-19 periods

Type of HAIs	Pre COVID (*n* = 321) Frequency (%)	During COVID (*n* = 269) Frequency (%)	Percentage change	*p* value*
Ventilator-associated pneumonia (VAP)	3 (0.9)	3 (1.2)	33.33	0.78
Pneumonia (PNEU)	26 (8.1)	19 (7.4)	-8.64	0.75
Urinary tract infection (UTI)	5 (1.6)	3 (1.1)	-31.25	0.69
Surgical site infection (SSI)	4 (1.2)	2 (0.8)	-33.33	0.57
CAUTI	6 (1.9%)	3 (1.1)	- 42.10	0.49
CLABSI	2 (0.6%)	2 (0.8)	33.33	0.82

*VAP* ventilator-associated pneumonia, *HAP* hospital-acquired pneumonia, *CLABSIs* central line-associated bloodstream infections, *CAUTIs* catheter-associated urinary tract infections, *UTI* urinary tract infection. ^*^: Pearson’s chi-square test

**Fig. 1:**
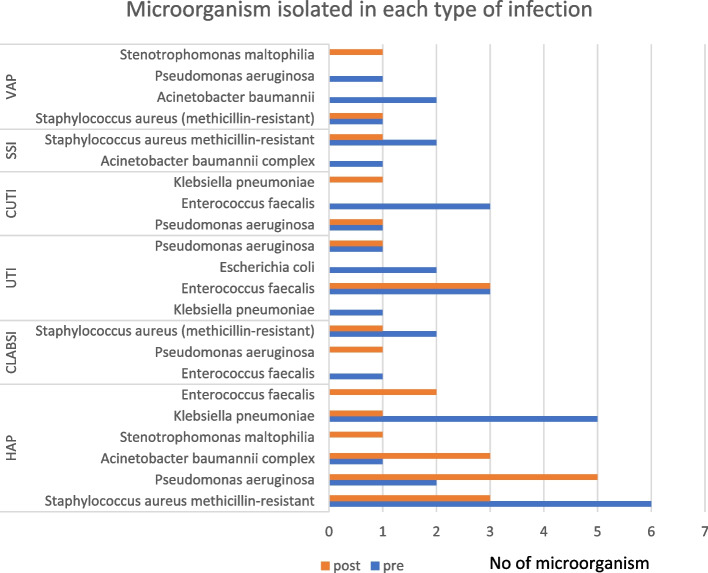
Distribution of pathogens isolates by type of hospital acquired infection

The HAI-related microorganisms did not significantly vary between the two times. A total of 35 microorganisms were isolated from HAIs in the period pre-pandemic and 26 microorganisms were isolated during the pandemic. HAIs were mostly caused by *methicillin-resistant Staphylococcus aureus* (MRSA) (28.2%) pre COVID-19 and 20.6% during COVID-19 with (*p* = 0.47). *Pseudomonas aeruginosa* (27.6%) was the most frequent organism in the during covid while, *Enterococcus faecalis* (17.9%) and *Klebsiella pneumonia* (15.3%) in pre COVID-19 (Table 3). By contrast, *Stenotrophomonas maltophilia* and *Enterobacter cloacae complex* were identified for HAIs during COVID-19 only with prevalence rate (6.9%). Another organism that detected in both periods was *Acinetobacter baumannii complex*. MRSA, *K. pneumonia* and* P. aeruginosa* were identified among patients with HAP. Whereas, MRSA was around 23.1% pre-COVID and 15.8% during COVID-19. While *K. pneumonia* was more detected in HAP patients in pre covid with parentage (19.3%). In contrast, *P. aeruginosa* was the most frequent pathogen causing pneumonia during COVID (26.3%). MRSA was remaining the main pathogen associated with other types of infections including VAP, CLABSI, and SSIs, during and pre COVID-19 (Fig. 1).

**Table 3:** Microorganisms isolated from patients with HAIs during both periods

Microorganism	Pre COVID-19 Frequency (%)	During COVID-19 Frequency (%)	*P* value*
*Klebsiella pneumonia*	6 (15.3)	2 (6.9)	0.28
*Pseudomonas aeruginosa*	5 (12.8)	8 (27.6)	0.12
*Escherichia coli*	2 (5.1)	0	
*Enterococcus faecalis*	7 (17.9)	3 (10.3)	0.41
*Enterobacter cloacae complex*	0	2 (6.9)	0.08
Staphylococcus aureus(methicillin-resistant)	11 (28.2)	6 (20.6)	0.47
Stenotrophomonas maltophilia	0	2 (6.9)	0.09
*Acinetobacter baumannii complex*	4 (10.2)	3 (10.3)	0.95

^*^: Pearson’s chi-square test

In the multivariate logistic regression analysis, both age and gender were found to be non-significant factors associated with HAI acquisition during both the pre-COVID-19 and COVID-19 periods (Table 4). As well as the comorbidities, including hypertension and diabetes mellitus, Conversely, the length of hospital stay emerged as a consistent and statistically significant risk factor associated with HAI acquisition in both timeframes, pre-COVID (OR = 1.27, CI 95%: 1.18–1.37, *P* < 0.001) and during COVID-19 (OR = 1.57, CI 95%: 1.27–1.94, *p* < 0.001). Regarding invasive procedures, urinary catheterization demonstrated a significant association with HAI acquisition before the pandemic (OR = 5.024, CI 95%: 1.52–18.01, *p* < 0.001*). In contrast, during the COVID-19 period, intubation and mechanical ventilation procedures were found to be significantly associated with an increased risk of HAI (OR = 11.10, CI 95%: 1.04–11.69, *p* < 0.001*) and (OR = 62.66, CI 95%: 2.45–16.00, *p* = 0.01*), respectively.

**Table 4:** Risk factors associated with HAI in the pre and during COVID -19 periods

Risk factors	Pre COVID 19			During COVID-19		
	OR	CI 95%	*P*	OR	CI 95%	*P* value
Age	0.97	0.93- 1.01	0.43	0.97	0.91–1.04	0.49
Gender	0.68	0.26- 1.77	0.19	2.36	0.37–19.49	0.36
Length of stay	1.27	1.18–1.37	**0.00**	1.57	1.27–1.94	**0.00**
Diabetes mellitus	0.84	0.30–2.31	0.74	5.64	0.77–40.03	0.08
Hypertension	0.76	0.23–2.47	0.65	0.16	0.00–3.10	0.22
Invasive procedure						
Intubation	2.02	044–9.04	0.36	11.10	1.04–11.69	**0.00**
Ventilation	2.58	0.40–16.42	0.31	62.66	2.45–16.00	**0.01**
Urinary catheter	5.024	1.52–18.01	**0.00**	0.75	0.41–13.70	0.84

Bold values are statistically significant (*P* < .05). *OR* odds ratio

## Discussion

HAIs have been identified as a leading cause of mortality and impose an enormous financial strain on the healthcare system [2, 24]. Adherence to infection control policies is an essential element in the management of HAIs [5]. Despite the extensive adoption of protocols aimed at mitigating the transmission of COVID-19 during the ongoing global pandemic [8, 17, 25]. This study found no statistically significant difference in the occurrence of HAIs between patients admitted to hospitals pre and during the onset of COVID-19 pandemic. A small reduction was observed in the rates of UTIs, SSIs, and HAP. The rate of UTIs demonstrated a decrease of 31.25%, whereas SSIs exhibited a decline of 33.33%. Furthermore, there was an observed decrease of 8.64% in HAP. These findings align with prior research that suggested that enhanced infection control strategies may not significantly impact HAI rates [26–28]. Unfortunately, the challenge of reducing HAIs has not received sufficient attention [24]. The heightened focus on managing COVID-19 pandemic has diverted resources away from traditional IPC measures in hospitals, which may have negatively affected HAI prevention [29, 30]. Additionally, variations in individual compliance with hand hygiene and personal protective equipment (PPE) use, as well as inadequate education, may have played a role [19, 25–28, 31]. In contrast, some studies have demonstrated the positive effects of improved IPC methods and strict adherence to established procedures in reducing HAIs during the COVID-19 pandemic [12, 14, 23, 32].

Regarding catheter-associated infections, the current research reveals a 42.10% reduction in the prevalence of CAUTIs, while rates of CLABSIs and VAP increased by 33.0% during the COVID-19 pandemic. These findings are consistent with previous studies that reported higher incidence rates of CLABSIs and VAP, possibly due to systemic stress, resource shortages, and a surge in critically ill patients [6, 33–35]. Improper PPE usage and reduced adherence to hand hygiene protocols among healthcare professionals may have contributed to the rise in CLABSIs and VAP [19, 31, 36]. Consequently, it is essential to implement targeted strategies aimed at reducing catheter-associated infections [37]. There is a pressing need to enhance evidence-based intervention approaches while strictly following standard transmission precautions [37, 38].

According to the study results, HAP was mostly caused by *Acinetobacter baumannii* complex and *P. aeruginosa* during the COVID-19 period [32, 39]. Notably, infection rates with *P*. *aeruginosa* were significantly higher (26.3%) than those caused by other bacteria. These organisms often exhibit antibiotic resistance and are associated with prolonged hospitalizations [4, 40]. In the pre-COVID-19, *Enterococcus faecalis* was frequently identified in UTIs, CLABSIs, and CAUTIs. However, the implementation of preventive measures during the COVID-19 pandemic may have led to a change in this pattern. *E. faecalis* was not detected in patients with catheter-related infections, including CAUTIs, CLABSIs, and VAP. The increased emphasis on hand hygiene during the COVID-19 pandemic is the most effective way to prevent the spread of E. faecalis [32, 41]. Therefore, alongside the initiatives implemented during the COVID-19 pandemic, continuous monitoring and prevention efforts are imperative [12, 23, 26].

The study revealed that the length of a patient's hospital stay has consistently been identified as a factor influencing the occurrence of HAIs [26, 42, 43]. Additionally, the use of invasive medical devices is another primary variable significantly contributing to the acquisition of HAIs in the two periods. In the pre-COVID period, urinary catheters were found to be highly relevant. However, during the COVID-19 pandemic, the utilisation of mechanical ventilation and intubation emerged as substantial risk factors for the development of HAIs [4, 30]. These findings underscore previous research findings that emphasise the impact of invasive medical procedures on the prevalence of HAIs [4, 38, 44]. Therefore, it is imperative to conduct a thorough assessment of clinical indications needing for the utilisation of these invasive medical devices [45]. This necessitates a heightened focus on both the initial insertion and ongoing maintenance of these invasive devices [37, 38]. This directive underscores the need for the implementation of a comprehensive and adaptable strategy aimed at effectively mitigating the risk of HAIs associated with invasive medical devices in healthcare settings [4, 32, 37].

The findings provide an overview and baseline awareness of HAIs rate in the hospital, which is essential for tracking future changes and assessing the effectiveness of infection control activities. However, there were no major differences between the two periods. These results may help to increase the effectiveness of the current infection control methods. Assist the hospital in improving its preparedness for any upcoming pandemic incidents.

As far as we are aware, this is the first study that discusses COVID's effect on the HAI rate in Malaysia. However, this study has several limitations. The major limitation of the study is the small sample size and the use of retrospective data analysis, which may limit the deep analysis to the risk factors and predictors of acquisition. Additionally, the generalizability of the study's findings to other healthcare facilities is limited as it was only carried out at one facility. Moreover, we didn’t evaluate compliance with infection control measures such as hand hygiene, using PPE. Further studies are needed to better understand the mechanisms underlying the insignificant decrease in incidence rates.

## Conclusion

While the COVID-19 pandemic did not yield a significant impact on decreasing the rate and types of HAIs between the two time periods studied, It is crucial to identify additional beneficial strategies in conjunction with highlighting the continuous necessity of maintaining strict compliance with IPC protocols.

## Data Availability

The datasets used and/or analysed during the current study are available from the corresponding author on reasonable request due to privacy reasons.

## Abbreviations

HAI: Hospital acquired infection
IPC: Infection prevention and control
VAP: Ventilator-associated pneumonia
CLABSIs: Central line-associated blood stream infections
CAUTIs: Catheter associated urinary tract infections
UTI: Urinary tract Infection
COVID-19: Coronavirus disease 2019
CVC: Central venous catheter
ICU: Intensive care unit
SSI: Surgical site infection
